# Non-Invasive Exploration of Neonatal Gastric Epithelium by Using Exfoliated Epithelial Cells

**DOI:** 10.1371/journal.pone.0025562

**Published:** 2011-10-18

**Authors:** Bertrand Kaeffer, Arnaud Legrand, Thomas Moyon, Anne Frondas-Chauty, Hélène Billard, Omar Guzman-Quevedo, Dominique Darmaun, Jean-Christophe Rozé

**Affiliations:** 1 Unité Mixte de Recherche-1280, Physiologie des Adaptations Nutritionnelles, Institut National Recherche Agronomique, Nantes, France; 2 University of Nantes, Hospital of Mother and Child, Nantes, France; University of Florida, United States of America

## Abstract

**Background & Aims:**

In preterm infants, exfoliated gastric epithelial cells can be retrieved from aspirates sampled through the naso-gastric feeding tube. Our aims were to determine (1) whether the recovery of exfoliated cells is feasible at any time from birth through the removal of the nasogastric tube, (2) whether they can be grown in culture *in vitro*, and (3) whether the physiological state of exfoliated cells expressing H+/K+ -ATPases reflects that of their counterparts remaining *in situ* at the surface of the gastric epithelium in neonatal rat pups.

**Methods:**

In infants, gastric fluid aspirates were collected weekly after birth or every 3 hours over 24-h periods, and related to clinical parameters (Biocollection PROG/09/18). In rat pups submitted to a single fasting/refeeding cycle, we explored circadian exfoliation with the cellular counter-parts in the gland. All samples were analyzed by confocal imaging and Enzyme-Linked Immunosorbent Assay.

**Results:**

Epithelial cells were identified by microscopy using membrane-bound anti-H+/K+ ATPases antibody, assessed for nucleus integrity, and the expression of selected proteins (autophagy, circadian clock). On 34 infants, the H+/K+ -ATPase-positive cells were consistently found quiescent, regardless of gestational age and feeding schedule from day-5 of life to the day of removal of the naso-gastric tube. By logistic regression analysis, we did find a positive correlation between the intensity of exfoliation (cellular loss per sample) and the postnatal age (p<0.001). The H+/K+ ATPase-positive cells established in culture retained the expression of a biomarker of progenitor status (Pouf5F1-Oct4). In rat pups, the expression pattern of Survivin in H+/K+ ATPase-positive exfoliated cells paralleled that observed in cells remaining at the surface of the gastric gland.

**Conclusions:**

Tracking parietal cells can improve clinical monitoring and understanding of the autophagic death via the phosphatidylinositol 3-kinase/Akt/survivin pathway.

## Introduction

Exfoliation has been described as an active biochemical process, highly context-dependent and linked to epithelium homeostasis [1], [2], [3], [4]. It is believed that epithelial cells, loosing contact with companion cells as well as extracellular matrix, enter anoikis [5]. The detachment of epithelial cells triggers both pro and antiapoptotic signals, such as nuclear factor kappa-B and inhibitor of apoptosis protein family members; these antiapoptotic mechanisms presumably delay the onset of apoptosis and allow cells to survive [6], [7]. The balance between these signals and the duration of detachment further determine the ultimate fate of these cells. Antiapoptotic signals presumably delay the onset of anoikis, allowing cells to survive provided they can reestablish contact with extracellular matrix in a timely manner [8]. Loss of extracellular matrix contact induces autophagy in normal epithelial cells; autophagy promotes the survival of detached cells during both anoikis and lumen formation in 3D epithelial cell culture [9], [10]. Under these assumptions, exfoliation may be understood as a natural process to remove external cells from the luminal surface of an epithelium. Consequently, exfoliation has a physiological role in the architecture of epithelium by allowing the formation of a lumen and we can surmise by providing sufficient flexibility to preserve the physical integrity of epithelia and allow its growth. By loosing contact with the original mucosa, exfoliated epithelial cells have to activate autophagy as a survival mechanism to endure starvation. Starving cells are degrading cytoplasmic material to generate both nutrients and energy [11]. Aoyama et al (2008) have shown on a rat model that the parietal cells are exfoliated into the gastric pit of isolated rat gastric mucosa after stimulation of acid secretion or under re-feeding conditions [2]. The renewal of pit parietal cells of the gastric glands is of potential interest to monitor the response of gastric epithelium to a stimulation by food intake or pharmacological agents. In premature infants receiving enteral nutrition, gastric fluids are routinely aspirated (and discarded) every 3 hours in neonatal intensive care unit to ensure adequate gastric emptying and the tolerance to enteral feeding. Exfoliated epithelial cells therefore can be isolated from such samples^3^. Our objective was to determine ***(1)*** whether the recovery of exfoliated cells is consistently feasible at any time from birth until the time of removal of the nasogastric tube, and ***(2)*** whether exfoliated cells found positive for H+/K+ -ATPase are in a physiological state comparable to their counterparts remaining *in situ* at the surface of the gastric epithelium.

In this paper, we report analyzes performed on samples from 34 infants to trace specifically exfoliated H+/K+ ATPase-positive cells. Such cells were consistently found in a state of quiescence from the 5^th^ day of life until the time of removal of naso-gastric tube (Day-30), regardless of gestational age and feeding schedule. In rat pups, exfoliated cells and cells within the gastric gland expressing H+/K+ ATPases, had comparable levels of expression of Survivin, a key member of the anti-apoptotic protein family.

## Results

### Exfoliated H+/K+ ATPase-positive cells of preterm infants retain a biomarker of progenitor status in culture

**Figure 1:**
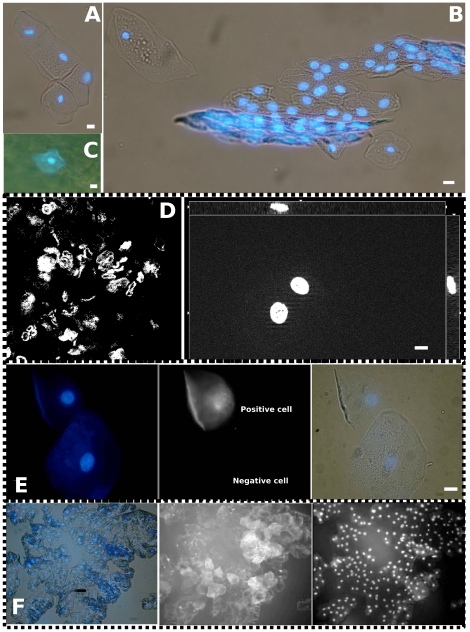
Morphology of H+/K+ ATPase-positive cells from preterm infants, freshly isolated and in primary cultures. A. B. Typical freshly isolated exfoliated cells from Day-5 to Day-36. C. Epithelial cell adherent to external wall of nasogastric tube. D. Samples obtained before Day-5 were mostly made up of cellular aggregates showing damaged nuclei (left insert). Later, all preparations contained epithelial cells showing quiescent nuclei (right insert). E. Pouf5-F1-Oct-4 positive and negative cells at inoculation for primary culture (center panel), nuclei are shown on left panel and nuclei inside cytoplasm shown on right panel. F. A colony of growing epithelial cells harboring Pouf5-F1-Oct-4 biomarker of progenitor status (center panel). Nuclei inside cytoplasm shown on left panel and nuclei staining by Hoechst on right panel. White bars stand for 10 µm.

To improve the yield of epithelial cells isolated by our previously described procedure [3], we recommend the current procedure which increases the yield of exfoliated cells by a tenfold factor (5,000 cells per sample instead of 500) while preserving the typical epithelial morphology of most cells (**Figure 1 A and B**). We also observed the presence of epithelial cells adhering to the plastic orogastric tube used to feed the babies (**Figure 1 C**). Samples obtained before Day-5 were mostly made up of cellular aggregates showing damaged nuclei. Later, all preparations contained typical epithelial cells showing quiescent nuclei (**Figure 1 D**). After isolation from gastric fluid aspirates of four preterm infants, colonies were readily obtained a few hours after inoculation. Inoculations were performed in P-6 or P-24 on tissue-culture treated plastics. We did not use any specific coating to improve cell adherence. Tissue culture medium was changed every third day. Typical densities were between 1,000 to 5,000 cells per cm^2^. In the inoculate, every other cell was expressing Pouf5F1Oct4 biomarker suggesting their progenitor status (**Figure 1 E**). After 2 days in culture, most cells were positively labeled by anti- Pouf5F1Oct4. Primary cultures could be maintained for 16 days (**Figure 1 F**) before microbial submersion.

### Quality and Intensity of exfoliation over one month of hospitalization

**Figure 2:**
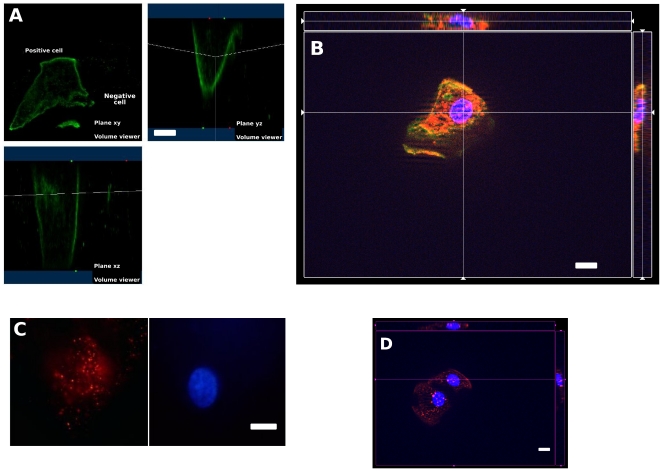
3D-view of exfoliated epithelial cells of preterm infant showing membrane-bound labeling of H+/K+-ATPases (A, B, in green), and intact nuclei labeled by Hoechst (in blue). The cytoplasmic expression of Survivin (B, red), LC3-b (C, red) and CLOCK (D, red). White bars stand for 10 µm.

Microscopic examination allowed to circumvent the inherent heterogeneity of clinical material by relating all results to the single cell and to easily distinguish quiescent from apoptotic cells [3], [12]. As shown in **Figure 2**, membrane-bound labeling by anti-H+/K+ ATPase antibody was clearly obtained with only 37% (+/−4.5%, n = 113) of the cell population which was positively labeled along with the expression of survivin, LC-3-b and CLOCK. Among 50 epithelial cells, 71% (+/−6.4%) were positively labeled by anti-survivin antibody. We did find only a weak labeling by anti-SLC-26A7 antibody suggesting that the proteins were rapidly recycled after the loss of cell-to-cell contact. Under microscope, we performed double blind enumeration by two independent investigators and found around 10,000 cells per gastric residual fluid aspirate (minimum: 2,000 – maximum: 15,000). The average size of quiescent nuclei was 10±4 µm (n = 50) compared with 14 µm±1.7 µm for Caco-2 cells, and 11±2 µm for exfoliated buccal cells from a healthy adult volunteer. The maximal cytoplasmic length of epithelial gastric cell of infants was of 37±2 µm, compared with 66±2 µm for epithelial buccal cells.

On 178 analyzed samples from 34 infants (**Table 1**), we have selected 72 samples obtained from 33 infants to create a database and explore the relations between clinical parameters and the intensity of exfoliation expressed as a semi-quantitative scale. As shown in **Table 2**, we found a highly significant effect of post-natal age and enteral volume on the intensity of exfoliation (p = 0.001). Other clinical parameters like post-conceptional age, gestational age, and weight at birth or at sampling day were all slightly significant (p<0.05). The z scores were non-significant (p = 0.36). We did not find any effect of gender (p = 0.65), of feeding with Mother's milk (p = 0.40) or with milks collected from the lactarium facilities (p = 0.33). However, a statistical trend was found with infants fed with Preterm milk formula (p = 0.058). On these data, we selected 4 explicative variables ((enteral volume, post-natal age, post-conceptional age, and preterm milk formula) and realized a logistic regression analysis to explore any difference between samples with low intensity of exfoliation (below or equal to 2) and samples with high intensity of exfoliation (higher or equal to 3). On **Table 3**, the Wald test indicated that 2 variables were explicative: post-natal age (calculated on 7 days; p = 0.016), post-conceptional age (p = 0.026). From database analysis, we found that 6 infants (sex ratio: 1) were fed only on Preterm milk formula. They had a higher intensity of exfoliation (Mean Intensity: 3.09, Maximum: 5, Minimum: 1, on 11 samples) than the 26 infants who were fed with breast milk or who received the milk of another mother (Mean Intensity: 2.03, Maximum: 6, Minimum: 0, on 53 samples).

**Table 1:** Distribution of patients according to gestational and post-natal age with the number of patients per analysis performed on gastric residual fluid aspirates collected from neonates (25–32 weeks).

Gestational age (weeks)	Number of patients (Sex ratio)	Number of samples per type of analysis		
		Culture & immunofluorescence	Immunofluorescence (before D-5/after)	ELISA
25	2 (1)	-	3 (0/3)	24
27	2 (1)	-	4 (0/4)	34
28	8 (1)	-	29 (5/24)	23
29	9 (1.7)	1	10 (3/7)	3
30	3 (0.5)	-	5 (1/4)	-
31	5 (1.5)	3	10 (4/6)	17
32	5 (0.7)	-	11 (0/11)	1
Total	34 (1.06)	4	72 (13/69)	102

Note that 13 samples were examined under microscope before 5 days of life. On 178 analyzed samples, we created a database of 72 samples tracing clinical information and biological analyzes useful for multifactorial analyzes on 33 infants (**Data S2**).

**Table 2:** Clinical parameters of the preterm infants.

	Intensity of exfoliation					p
	0	1	2	3	>4	
**Post-natal age (days)**	1.00 (0.89/6)	18.33 (13.53/21)	15.11 (10.00/18)	18.00 (8.72/19)	32.38 (9.77/8)	**0.001**
**Age post-conceptional (days)**	30.33 (1.63/6)	31.19 (2.29/21)	31.17 (1.58/18)	32.52 (2.27/19)	32.62 (1.30/8)	**0.044**
**Gestational age (weeks)**	30.00 (1.26/6)	28.62 (1.94/21)	29.06 (1.98/18)	29.95 (1.71/19)	28.00 (0.00/8)	**0.036**
**Birth weight (g)**	1403.33 (386.87/6)	1167.71 (334.73/21)	1295.00 (394.07/18)	1501.84 (424.06/19)	1144.38 (143.29/8)	**0.044**
**Weight at sampling day (g)**	1343.33 (426.95/6)	1361.00 (368.54/20)	1395.82 (289.55/17)	1730.89 (604.77/19)	1649.37 (246.60/8)	**0.043**
**Birth weight Z-scores**	−0.5478 (1.12809/6)	−1.07 (0.70132/20)	−0.8716 (0.61411/17)	−0.7217 (0.56185/19)	−0.8909 (0.29333/8)	**0.360**
**Enteral volume (ml/day/Kg)**	33.18 (24.98/4)	104.48 (62.69/17)	132.93 (46.00/17)	139.79 (47.03/17)	160.04 (8.85/8)	**0.001**

**Table 3:** Logistic regression analysis with 4 variables.

	aOR	95% CI	p
**Enteral volume (by 100 ml/day/Kg)**	1.9	[0.8–4.3]	0.143
**Postnatal Age (by 7 days)**	2.8	[1.2–6.7]	0.016
**Postconceptional age (by week)**	0.3	[0.1–0.9]	0.026
**Milk formula**	17.8	[0.7–399.3]	0.072

aOR: adjusted Odds ratio, CI: confidence interval.

The test of Wald suggest that 2 variables are explicative: the postnatal age and the postconceptional age.

**Figure 3:**
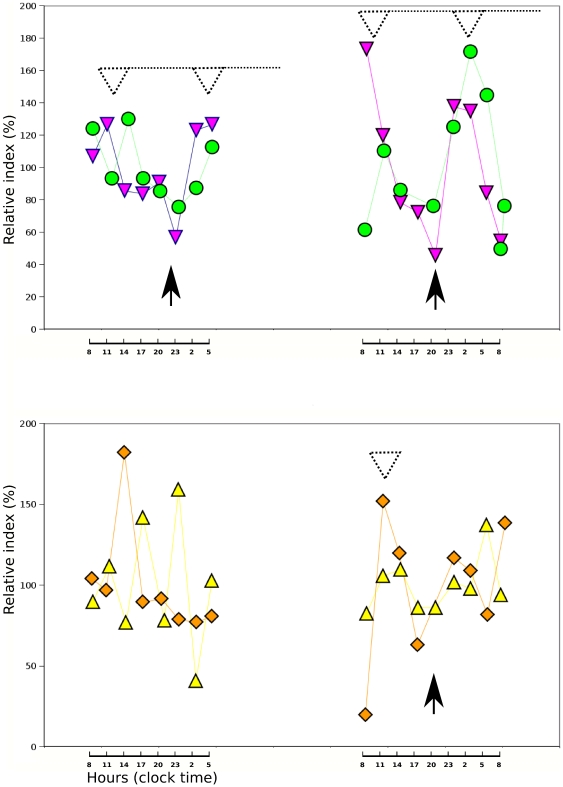
Kinetics of exfoliation of H+/K+-ATPase-positive cells over 24-hours followed by ELISA. Four infants were sampled every 3 hours on two consecutive 24-h cycles. Infant 1(inverted triangle) and 3 (circle) had similar profiles for both cycles. Infant 3 (triangle) and 4 (diamond) were comparable for the second 24-h cycles. Note that infant 4 (diamond) was unfed but showing low level of cellular loss. White dotted arrow heads indicate acrophases and black arrow heads indicate bathyphases.

**Kinetics of exfoliation from the gastric epithelium of preterm infants over two 24-hour cycles:** Serial collection of aspirates over 24-hours was performed to determine whether any fluctuation occurred in the rate of cellular loss in infants fed with a regular milk formula, and verify that exfoliation did occur even in infants fed intravenously and without any enteral feeding. Significant differences over time were found at a 5% level by ANOVA (**Figure 3**, **Figure S1 and Data S1**). The oscillation of CLOCK expression in the nuclei of buccal cells recovered by mechanical exfoliation can be compared with these data (**Figure S1**).

### H+/K+ ATPases- positive cells from the surface of the rat gastric gland or freshly exfoliated are expressing similar levels of survivin

**Figure 4:**
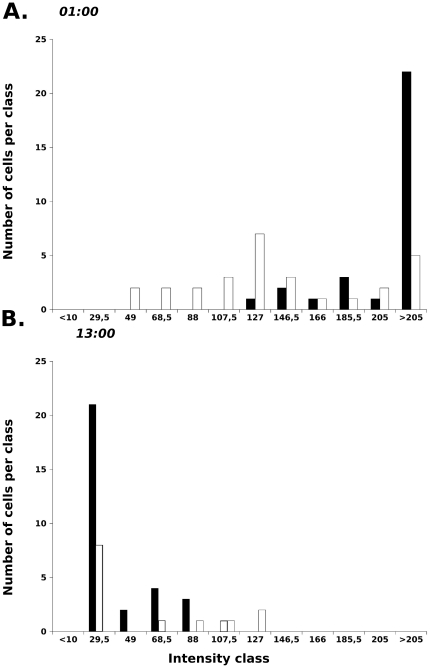
Distributions of intensity of labeling by anti-Survivin antibody of gastric cells after exfoliation (white bar) or within the gland (black bar) on hand-picked cells with a quiescent nucleus and a membrane labeling by H+/K+-ATPases-antibody. The distributions of intensities of cells within the gland were widely different at 13H00 and 01H00 (note black bar positions on A and B). The level of survivin labeling of H+/K+ ATPase-positive cells by exfoliated cells and cells within the gastric gland, were similar at 01H00 (A) and 13H00 (B).

The amount of H+/K+ ATPase-positive exfoliated cells in gastric contents of rat pups and cell supernatants of cultured gastric glands is higher at 13:00 than at 01:00 (**Figures S2 and S3**). On samples of 30 hand-picked cells with a quiescent nucleus and a membrane labeling by H+/K+-ATPase-antibody, the distribution of labeling intensity by anti-Survivin antibody differed widely between pups sampled at 13H00 and 01H00 (**Figure 4 A and B**, solid black bars).

The level of survivin labeling of H+/K+ ATPase-positive exfoliated cells and cells within the gastric gland, were similar as shown **figure 4** on data obtained from a rat pup with a bolus of saline solution. At 01H00 (**Figure 4 A**), 78% of cells exfoliated in vitro were in the same category as cells stained *in situ* within the gastric gland (confidence interval: 64,28%–91,72%, at the p = 0.05 level n = 30). At 13H00 (**Figure 4 B**), 84% of cells exfoliated in vitro were in the same category as cells stained *in situ* within the gastric gland (confidence interval: 75,6–92,4%). The level of LC-3-b or CLOCK labeling of H+/K+ ATPase-positive exfoliated cells and cells within the gastric gland were determined at 13H00, with 90% (9%) and 100%, respectively.

## Discussion

The current study demonstrates that H+/K+ -APTase – positive quiescent epithelial cells are consistently recovered from any gastric fluid aspirate obtained from preterm infants from the 5^th^ day of life, regardless of sex and gestational age between 25 weeks and 32 weeks.

A major concern with the use of exfoliated epithelial cells is to be able to trace their origin in order to rule out any contamination from the environment (i.e. exfoliated epithelial cells from the mother's mammary gland). We used the expression of H+/K+ ATPase, or gastric pump, to minimize the risk of detecting epithelial cells of the mother's mammary gland. We therefore chose to trace solely cells expressing membrane H+/K+ -ATPases because such cells ***(1)*** belong to the pit parietal cell lineage, ***(2)*** can be traced by their clear-cut membrane labeling by anti-H+/K+ -ATPases both on infant and rat pup cells. Our results are in favor of a highly specific detection as infants who received only Preterm milk formula are loosing more cells expressing H+/K+-ATPase than infants who received breast milk or milk from another mother; moreover unfed infant shown on **figure 3** is showing low level of cellular loss.

Epithelial stem cells in the neck region of the gastric gland are responsible for the continuous renewal of the epithelium through generation of multiple gastric cell lineages that populate the epithelium. Some gastric progenitor cells could function as sensors of damage, or reservoirs with high proliferative potential that can regenerate portions of the gastric mucosa after injury [13], [14], [15]. In our study, the integrity of nuclei was systematically ascertained by Hoechst staining to select for quiescent cells expressing H+/K+ -APTases. To prove that exfoliated epithelial cells recovered from infants were viable, we grew these cells in explants in culture. The high level of expression of Pouf5-F1-Oct-4 by approximately every other exfoliated cell (**Figure 1-E**) suggests that the postnatal development of gastric gland in preterm infant is similar to that described in neonatal mice [12]. In addition, most of these quiescent epithelial cells can readily grow in culture suggesting that detachment-induced autophagy contributes to the viability of these cells. These data can be compared with those on human mammary epithelial cells which retain some clonogenic potentials after detachment and die after 24–48 hours in the environment [10]. Quiescent exfoliated epithelial cells can be isolated from different body fluids (breast milk, urines, digestive fluids). They are believed to enter rapidly in anoikis after exfoliation. In cells having lost contact with tissue structure, autophagy corresponds to the recycling of cellular material as well as to the cell capacity to mobilize reserves during periods of starvation. Autophagy is viewed as a survival mechanism during fasting periods; autophagy must, however, be controlled/stopped at the whole organism level to prevent self-digestion. Anoikis can be considered as an autophagic state promoting epithelial cell survival after a timely loss of contact with extracellular matrix and cell neighbors [8]. Further molecular work on gastric exfoliated cells of preterm infants would be needed to delineate anoikis from apoptosis. A functional assay (like using some pHi indicator with a stimulation by histamin) designed directly on the cell suspension right after the isolation process would be highly desirable but difficult to perform due to the small number of cells and the need to cross-check the cellular phenotypes. Phenotype and physiological profiling by a transcriptomic approach (like in [1] and [3]) will help to cross-check the diversity of these H+/K+-ATPase-positive cells. Our data (**Figure 1-F**) nevertheless pave the way to the selection of primary cell cultures derived from human preterm gastric mucosa. We cannot trace exactly the cellular position in the infant's stomach but it would be of crucial interest to know whether the exfoliated gastric cells can be used to build long-lived gastric units in vitro [16]. After solving the problem of heavy bacterial contamination of cells, we believe that it would be informative to design a functional assay on these cell cultures to ascertain which part of the stomach (cardia, corpus, pylorus) they are their representative?

Our study was undertaken to explore the time course of exfoliation from birth to the time of removal of naso-gastric tube. From samples obtained between the 5^th^ day of life up to naso-gastric tube removal (30–36 days later), we did not find any effect of the gestational age nor of gender but we found a strong effect of post-natal age and enteral volume (**Table 2**). Consequently, the gastric epithelium is mature and functional within few days after birth whatever the term of birth between 25 to 32 weeks. A non-significant influence of milk formula on the intensity of exfoliation has been found (p = 0.058) calling for future works on the influence of feeding schedule or of various milk formulas on gastric epithelium's exfoliation. According to current works on the nutritional induction of exfoliation by nutrients made on rodents, gastric epithelial cell exfoliation may represent a direct way to appreciate the nutritional or pharmacological stress induced by a substance on the epithelium's homeostasis.

As collection of gastric residual fluid aspirates is part of the nursing care routine performed every three hours in many neonatal intensive care units (NICU), the technique is highly relevant for time series experiments in chronobiology. Rivkees et al (2004) have shown that the Rest-Activity patterns of premature infants are regulated by cycled lighting with an organization detectable at 34 weeks [17]. Kaeffer et al (2007) have shown that clock compounds are expressed by gastric exfoliated cells at 28 weeks [3]. Recently, Chen (2010) found that Bmal1 and cryptochrom transcripts are present in peripheral blood cells at 30 weeks, however, no oscillation of these transcripts was found [18]. However the network of circadian clocks in adult mammals is partly autonomous and partly driven by central clock located in suprachiasmatic nucleus (SCN) of hypothalamus (i.e. the one in the liver is believed to be entrained both by SCN and feeding cues [19]). Stomach is part of the gastrointestinal sphere and believed to be entrained by both SCN and feeding cues. Our data on **figure 3** suggest that exfoliation is following a circadian rhythm according to data on adult rat hepatocytes [20] and according to our data recorded at noon and midnight on rat pups (**Fig. 2** **-S, Data S1**, **Figure 4**). However we cannot completely rule out the possibility of a mechanical abrasion of the surface of the gastric epithelium by the pumping device giving a false circadian rhythmicity of exfoliation. We may only speculate that if quiescent epithelial gastric cells are retaining fully functional clocks, they may retain chronobiological information consistent with their time at exfoliation and subsequent 3-hour cell survival out of the organism. The induction of gastric cell exfoliation by nutrient cycle on lactating rat pups can be used in the future to address questions about the stability of clock information during anoikis. In our hands, exfoliated cells expressing H+/K+ ATPases can be recovered from gastric fluids of rat pups in a physiological state comparable to their counter part cells remaining *in situ* at the surface of the gastric gland (**Figure 4** **, Figure S3, Data S1**).

In cancer cells like Prostate cancer PC3 cells, the balance between survival and death is attributed to an interaction between MAP-LC3-b and survivin [21]. In our work, exfoliated cells expressing H+/K+ ATPases were found to constantly express high levels of survivin (**Figure 2 B**) calling for future investigations to correlate by Western blot analysis the direct interaction between MP-LC3-b and survivin. We reported recently that perinatal denutrition in rat pups has a wide impact on the metabolism and behavior of young rats [22]. Direct exploration of autophagic and circadian clock machinery on exfoliated epithelial cells isolated from preterm infants of different nutritional status will allow to detect early deficiency which might be corrected by appropriate nutritional or pharmacological manipulations.

In conclusion, H+/K+ -ATPase positive cells harboring markers of a progenitor status can be recovered from gastric fluid aspirates in preterm infants whatever the term or the postnatal age to measure the expression of specific genes of interest and explore non-invasively the functioning of neonatal gastric epithelium.

## Materials and Methods

### Ethics statements

Concerning studies on preterm infants, all samples **in** **Table 1** were collected from preterm infants in the Neonatal Intensive Care Unit at the “Hôpital de la Mère et de l'Enfant” at Nantes, France. The protocol was approved by the Nantes Hospital Ethics Committee (PROG/09/18). Written informed consent were obtained from both parents (or guardians) within PROG/09/18 and written informed consent was independently obtained from the healthy volunteer sampled for inner cheek mechanically exfoliated epithelial cells.

Concerning studies on rat pups, animal experiments were realized according to the rules of the Nantes animal experimental unit (in compliance with the European Communities Directive of 24 November 1986 (86/609/EEC) and the Principles of laboratory animal care (NIH publication no. 85-23, revised 1985). The protocol was strictly non-invasive for rat pups and mothers and as such was approved under the number P-2010-01 by the local review board of UMR-1280 animal husbandry (members: Martine Champ, Guillaume Poupeau, Franck Doulay, Isabelle Jicquel). Animals were euthanized by carbon dioxide exposure.

### Preparation of exfoliated epithelial cells

Fluid aspirates of preterm infants or gastric contents of rat pups were stored at −70°C and processed within one month of sampling using identical procedure and buffers to recover exfoliated epithelial cells. The procedure to obtain gastric fluid aspirates is standard in our neonatalogy unit. Preterm infants equipped with a nasogastric tube are fed over 3-hour periods, using, for instance 30 mL of milk slowly instilled by a syringe pump device into the stomach lumen. At the end of each 3-hour period, the nursing staff disconnects the device and collects empties the infant's gastric residues by manual aspiration using a sterile syringe connected to the nasogastric tube. Undigested milk and gastric fluids are then removed by a slow depression of the syringe. The gastric fluid aspirate (0.5–1.5 ml) is transferred into sterile plastic tubes for immediate processing or direct storage at −70°C. Briefly, gastric samples were thawed and diluted out in 15 ml EDTA/DTT buffer (3 mM EDTA and 0,05 mM DTT in PBS0 during 2 min on ice. Cellular material was pelleted by centrifugation at 2000 rpm during 3 min. Supernatant was discarded and cellular pellets were resuspended into 12 ml PBS0, vigorously pipetted six times and pelleted again by centrifugation at 2000 rpm during 3 min. Cellular pellets were either stored at −70°C for ELISA or fixed and kept at 4°C up to microscopic analysis. Fixation was performed with freshly made formaldehyde 4% solution for one night and replaced by ethanol 70% at 4°C for storage up to labeling after rehydration, or by paraformaldehyde 4% saline solution after rinsing by PBS0 and stored at 4°C. Times series of exfoliated cells from the inner cheek of an adult volunteer were obtained every 6 hours over 24 hours to trace the nuclear expression of CLOCK factor and to follow LC-3-b expression over 3 hours of fasting by maintaining cells in PBS-0 buffer. Colon cancer Caco-2 cells were also loaded into freshly collected gastric fluid aspirates to assess the efficiency of recovery.

### Selection of biomarkers, source and specificity of primary antibodies

H+/K+ ATPase is specifically expressed by pit parietal cells of the stomach [23] and can be detected with mouse monoclonal antibodies as well-defined membrane labeling (Abcam (ab-2866)). The H+/K+ ATPase, or gastric pump, share structural similarities like an alpha and beta subunit, with the ubiquitous Na+/K+ ATPase. The monoclonal antibody that we used is specific for the beta-subunit which may play a role in maintaining the structural and functional integrity of the complex. To our knowledge, expression of H+/K+ ATPase has never been reported to occur in exfoliated epithelial cells from the mammary gland. Survivin, a member of the inhibitors-of-apoptosis protein (IAP) family, has been described as expressed in gastric parietal cells of adult rats and humans [24]. *In situ* detection of microtubule-associated protein light chain 3b (LC3b) by primary antibodies (Santa-Cruz (sc-28266) has been recommended when this protein constitutive of the autophagosome is overexpressed during progressive autophagy [25]. However, autophagy has been demonstrated to occur in vivo in the surface epithelial cells of neonatal small intestine of piglets [26]. Rabbit polyclonal anti-survivin (Santa-Cruz, sc-10811) antibody was used to characterize non-apoptotic status of our cells and the fraction of highly viable cells with a status of progenitor cells [27] along with the expression of POUF5F1-OCT4 [28] (Sigma – P0873 and Abcam, ab-49091). Determination of cytokeratin 18 expression (a marker of epithelial origin) was also performed on dot blot (Santa-Cruz, DC-10, sc-6259 and H-80, sc-28264). SLC-26A7 is expressed mainly by epithelial cells attached to the tissue [29] (detection by Santa-Cruz sc-53960). In addition, we targeted CLOCK and Period1, both involved in circadian rhythms. The transcription factor CLOCK was detected by antibodies from Abcam ab3517 and Santa-Cruz, sc-25361. CLOCK harbors a Histone-acetyl-transferase activity, and histone acetylation is thought to play a key role in the effects of early nutrition on gene expression, possibly mediating the long-term effects of early nutrition (nutritional imprinting). Period1 is another immediate response gene involved in the quick resetting of the circadian clock (Santa-Cruz sc-25362). Antibodies were used in serial dilution in confocal microscopy or ELISA according to manufacturers' requirements.

### Primary culture of exfoliated epithelial cells from four preterm infants

Gastric cells were prepared from freshly sampled gastric fluid aspirates and inoculated in DMEM with 20% fetal calf serum, 2 mmol/L L-glutamine, penicillin-streptomycin (Invitrogen, Cergy Pontoise, France), amphotericin-B, and gentamicin (Sigma-Aldrich, St-Quentin Fallavier, France). We used P-24 (Nunc® UpCell^™^ Surface cell culture multidish, 24 wells) and P-6 (Nunc® Nunclon Vita Multidish, 6 wells) tissue culture plates. Tissue culture plates were maintained in a humidified incubator at 37°C under 5% CO_2_. Trypan blue exclusion test was performed to check for cell viability.

### Nutritional induction of exfoliation in rat pups

Female Wistar rats were housed either in a 12∶12 light∶dark (LD) or dark∶light (DL) cycle for a week before impregnation. Subgroups of 6-day old (n = 40) and 12-day old rat pups (n = 16) were separated for 5 hours from their mother but kept in eye-contact in a container maintained in a 37°C-water bath. Pups were then re-united with their mothers and allowed to feed for one hour before sacrifice. Rat pups were sacrificed at 13H00 and at 01H00. Contents were recovered by soft massaging of the stomach and stored at −70°C. Stomachs were used either for in vitro exfoliation after rapid rinsing in 10 ml PBS0 or for immunohistochemical examination. In vitro experiments were performed after rat pups half-stomach was sectioned from cardia to pylorus, and immersed in DMEM-GlutaMax+10% fetal calf serum with antibiotics for one hour at 37°C under 5% CO_2_. Both halves of stomach were recovered with a pincer and gently rinsed in PBS0. The first half was fixed and placed in cassettes for cryosection, embedded in DHE polymer taking care to orient the lumen of the gland upward. The second half was placed overnight in Glucose : Paraformaldehyde solution (4%) for fixation. The next day, tissues were placed in cassettes for cryosection, embedded in DHE polymer taking care to orient the lumen of the gland upward. In order to realize immunostaining for microscopic examination, gastric sections were rapidly thawned and saturated for one hour at 4°C in PBS0+0,2% Bovine Serum Albumin fraction V (BSA). Primary antibodies were diluted in PBS0+0,2% BSA and incubated on sections overnight at 4°C in a special device. After 3 cycles of washing by PBS0, sections were incubated with secondary antibodies and Hoechst 33258. Exfoliated cells in supernatants were collected by centrifugation (2000 rpm, 3 min) and stored at −70°C or fixed by freshly made paraformaldhyde 4% saline solution.

### Immunocytochemistry and confocal imaging

Nuclear DNA was stained with Hoechst 33258 (Molecular Probes) or DRAC-5 (In Vitrogen) fluorochromes. Hoechst staining was systematically used to determine quiescent versus apoptotic nuclei. Apoptotic figures were rarely seen and not included in analysis. Cell and tissue preparations were mounted in Prolong Gold (In Vitrogen) and visualized under Zeiss apotome microscope or Leica confocal microscope. The intensity of labeling by the primary – secondary antibodies complex was normalized by the total surface of the cellular body at the best plane of acquisition by densitometry with ImageJ software. Cell morphology was compared with buccal epithelial cells of human adult exfoliated by swabbing or a colon cancer cell line (Caco-2 cells, purchased from the European Collection of Animal Cell Culture, UK: ECACC #860120). With rat pups, exfoliated cells and surface cells of the gastric gland harboring a quiescent nucleus, fixed as viable according to a high intensity of MitoTracker Far Red labeling [30], expressing a strong membrane-bound labeling with H+/K+ ATPases antibody were identified manually under microscope. The best plane of a stack of electronic sections was selected and quantified for the expression of survivin performed on independent preparations with proper corresponding controls. Incubations of primary antibodies were carried out overnight at 4°C. After 3 washing cycles with PBS0, cell preparations were incubated with Hoechst 33258 and secondary antibodies were either Goat Anti-mouse-Alexa-488 or Goat-anti-rabbit-Alexa-568 (both from Molecular Probes) during one hour at 37°C. After 3 washing cycles with PBS0, cellular preparations were mounted into ProLong Gold. With preterm infants, cells expressing clear membrane labeling by anti-H+/K+-ATPase antibody were selected to quantify the expression of survivin or LC-3-b or CLOCK. Preparations were observed under an Apotome-Zeiss microscope with Axiovision 4.3 software, colocalization and quantification were realized both under this system and with ImageJ 1.42. Image stack were captured with a 1 µm z-resolution and 0.31 µm per pixel as (x, y) resolution. Intensities of labeling expressed by every cell were arranged in classes of intensity to calculate the frequency by class (I. e. The number of cells belonging to every class gave the frequency).

### Enzyme linked-immunoassay (ELISA)

To prepare antigens, cellular pellets were lyzed by 3 cycles of freezing – thawing from −20°C to Room Temperature. An aliquote of lysates was resuspended in saturation buffer (0,5 M Na_2_CO_3_/NaHCO_3_, pH 9.6) and 100 µL were incubated overnight on Greiner P-96 microplates. Well contents were discarded and post-coated with 150 µL of PBS0+0.2% BSA for 2 hours at 4°C. Plaques were rinsed with 100 µL PBS0 and incubated overnight with primary antibodies diluted according to suppliers' requirements in PBS0+0.2% BSA, overnight at 4°C. After three washing cycles by PBS0+0,05% Tween-20, plaques were incubated with secondary antibodies either Goat Anti-mouse-biotin (Sigma, B-0529) or Goat-anti-rabbit-biotin (Vector-lab, BA-1000) at 1/1000 during 2 hours at 37°C. After 3 washing cycles, plates were incubated with Extravidin-horse-radish peroxidase (Sigma, E-2886, 1/1000) for 30 min at 37°C. After 3 washing cycles, plates were revealed by ABTS (Sigma A-9941), in citrate buffer PH 3 (Sigma) containing 2% hydrogen peroxide. Densitometry was measured with a spectrophotometer Bio-Tek at 405 nm. Data were expressed as a Relative Index (RI). RI = (Optical density corrected from background – Mean Optical density of the serie/Mean Optical density)×100 (expressed in percentage).

### Statistics and Database

On data obtained from immunofluorescence imaging, normality of distribution of the intensity of a specific labeling was tested on 30 cells at a 5% level according to Kolmogorov test. Clinical and biological measurements were organized into a database of 72 samples with clinical parameters and biological measurements to realize contingency and logistic regression analyzes. In this database, exfoliation was measured on every sample by concurring techniques (confocal imaging and/or ELISA). As one of our aim was to evaluate the number of H+/K+-ATPase-positive cells per sample, we created a semi-quantitative index. The intensity of exfoliation was defined as the cellular loss per sample. Logistic regression analysis was performed on 2 groups of intensities of exfoliation: low intensity from 1 to 3 and high intensity from 4 and higher. Statistical data obtained by ELISA were analyzed by ANOVA. All tests were two-tailed and the significance level was set at .05% level.

## Supporting Information

Figure S1 **Kinetics of exfoliation of H+/K+-ATPase-positive cells over 24-hours followed by ELISA.** Four infants were sampled every 3 hours on two consecutive 24-h cycles. Note that infant 4 was unfed the first nycthemer of sampling (orange diamond) but showing low level of cellular loss. Profiles were obtained in parallel preparations of H+/K+-ATPases detection, with antibodies against survivin (A, B), MAP-LC-3-b (C, D)), Pouf5-F1-Oct4 (E, F), PERIOD1 (G, H) and CLOCK (I, J). Note that by ELISA, we detect the antigens from any cellular phenotypes in the preparation along with cell-free antigens.
(TIF)

Figure S2 **Detection of biomarkers of exfoliated cells in cell culture supernatants or stomach contents.** The detection of biomarkers was done by ELISA on preparations of epithelial gastric cells exfoliated from gastric glands maintained in culture for 60 min (black symbols) or recovered from gastric contents (white symbols) of 12-day-old rat pups refed one hour before sacrifice. Note the similarity of antigen detection profiles of black and white symbols for all biomarkers. A significant difference was found between 13:00 and 01:00 series for all biomarkers by Student't test (p<0.05) except LC-3-b in contents, and CLOCK both in vivo and in vitro exfoliation.
(TIF)

Figure S3 **Gastric exfoliated cells of rat pups.** A. Typical exfoliated cells expressing membrane-bound H+/K+ ATPase (green, Alexa 488), showing a quiescent nucleus (blue, Hoechst). B. Survivin (red, Alexa 568). C. LC-3-B (red, Alexa 568). D. CLOCK (red, Alexa 568). E. Viable mitochondrial network in an exfoliated cell (yellow, MitoTracker Far Red). F. Surface epithelial cells of the gastric gland with viable mitochondries (yellow) showing a gradient of MitoTracker Far Red labeling with a maximum on the outside of the gastric sample and a minima inside the gastric sample.White bars stand for 10 µm.
(TIF)

Data S1 **Additional information on methodology and results.**
(DOC)

Data S2 **Raw data of clinical and biological variables measured on preterm infants.**
(XLS)
