# Supplementary figures and images for: Non-Invasive Exploration of Neonatal Gastric Epithelium by Using Exfoliated Epithelial Cells
Source: PLoS One. 2011 Oct 18;6(10):e25562. doi: 10.1371/journal.pone.0025562 (PMC3196493; doi:10.1371/journal.pone.0025562)

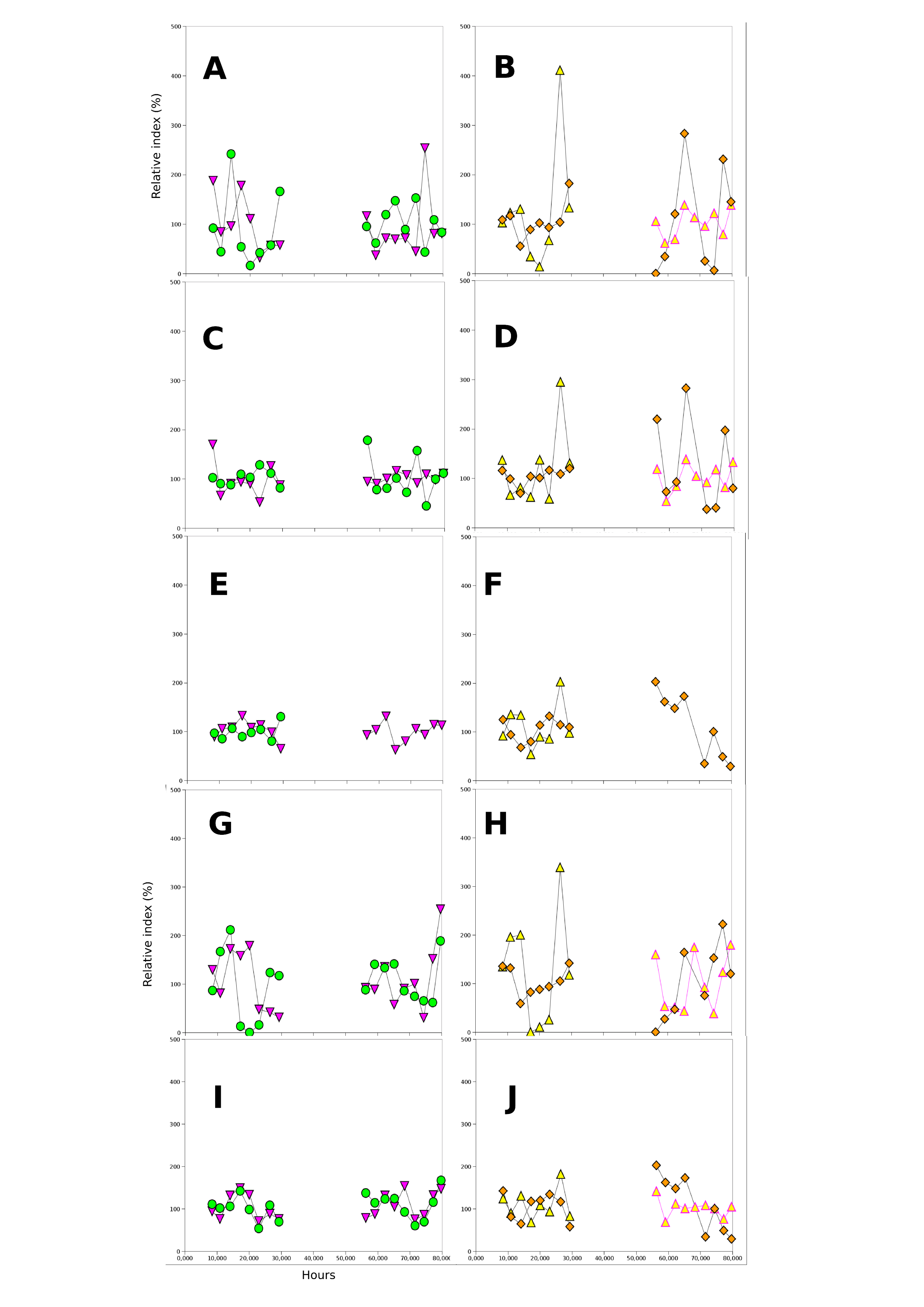

Supplement: Figure S1 — Kinetics of exfoliation of H+/K+-ATPase-positive cells over 24-hours followed by ELISA. Four infants were sampled every 3 hours on two consecutive 24-h cycles. Note that infant 4 was unfed the first nycthemer of sampling (orange diamond) but showing low level of cellular loss. Profiles were obtained in parallel preparations of H+/K+-ATPases detection, with antibodies against survivin (A, B), MAP-LC-3-b (C, D)), Pouf5-F1-Oct4 (E, F), PERIOD1 (G, H) and CLOCK (I, J). Note that by ELISA, we detect the antigens from any cellular phenotypes in the preparation along with cell-free antigens. (TIF) [file pone.0025562.s001.tif]

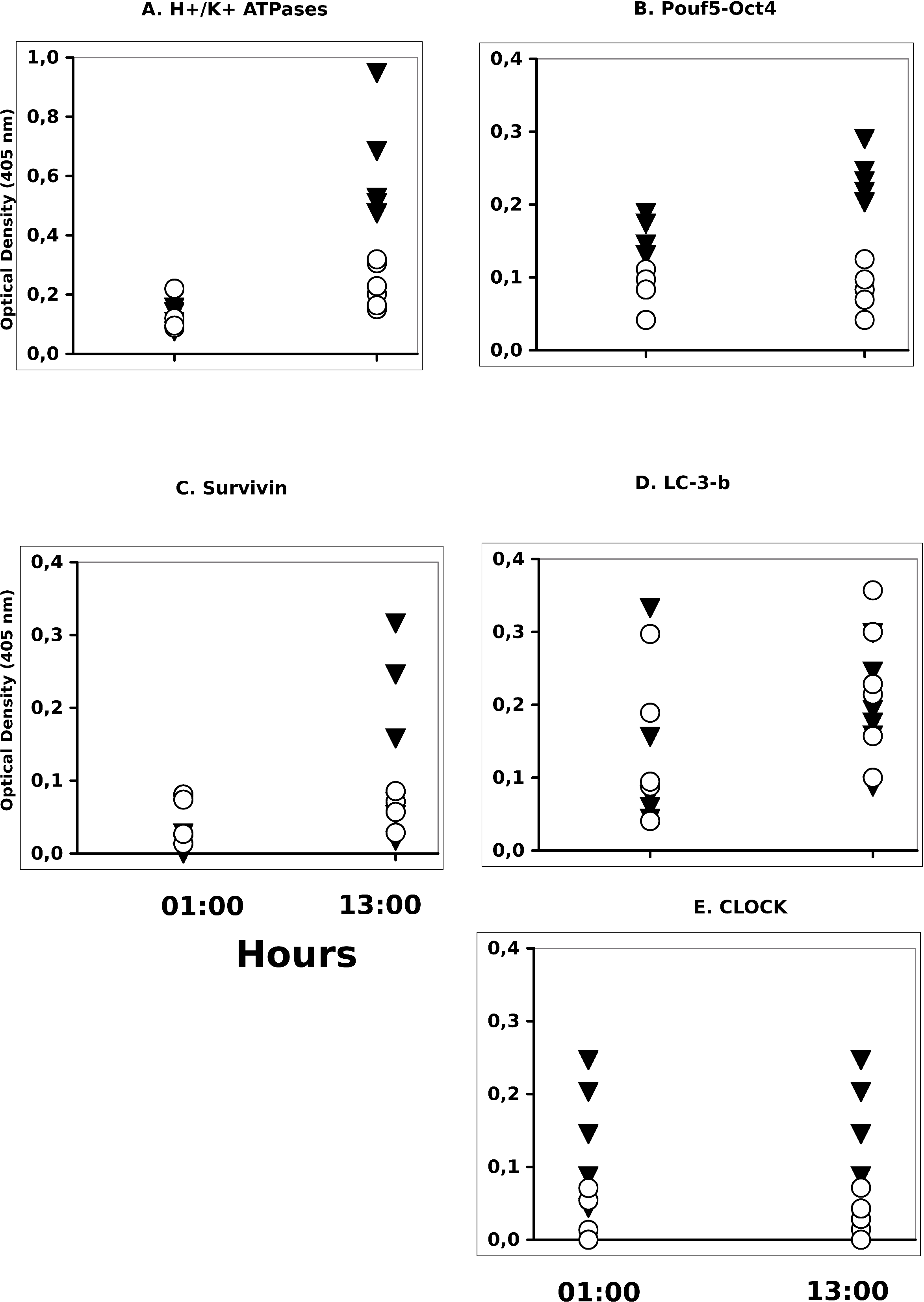

Supplement: Figure S2 — Detection of biomarkers of exfoliated cells in cell culture supernatants or stomach contents. The detection of biomarkers was done by ELISA on preparations of epithelial gastric cells exfoliated from gastric glands maintained in culture for 60 min (black symbols) or recovered from gastric contents (white symbols) of 12-day-old rat pups refed one hour before sacrifice. Note the similarity of antigen detection profiles of black and white symbols for all biomarkers. A significant difference was found between 13:00 and 01:00 series for all biomarkers by Student't test (p<0.05) except LC-3-b in contents, and CLOCK both in vivo and in vitro exfoliation. (TIF) [file pone.0025562.s002.tif]

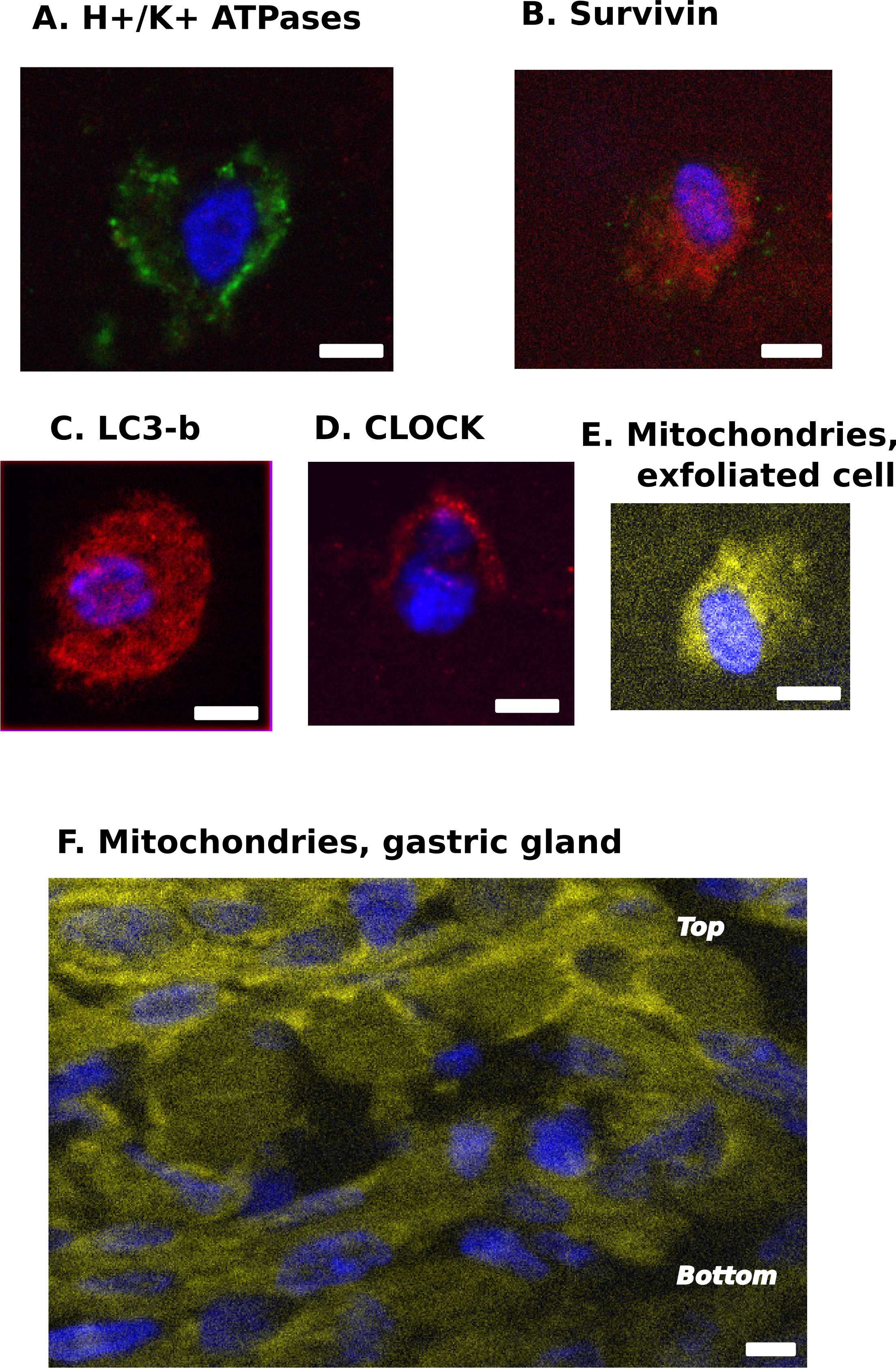

Supplement: Figure S3 — Gastric exfoliated cells of rat pups. A. Typical exfoliated cells expressing membrane-bound H+/K+ ATPase (green, Alexa 488), showing a quiescent nucleus (blue, Hoechst). B. Survivin (red, Alexa 568). C. LC-3-B (red, Alexa 568). D. CLOCK (red, Alexa 568). E. Viable mitochondrial network in an exfoliated cell (yellow, MitoTracker Far Red). F. Surface epithelial cells of the gastric gland with viable mitochondries (yellow) showing a gradient of MitoTracker Far Red labeling with a maximum on the outside of the gastric sample and a minima inside the gastric sample.White bars stand for 10 µm. (TIF) [file pone.0025562.s003.tif]
