# Supplementary material for: Non-Invasive Exploration of Neonatal Gastric Epithelium by Using Exfoliated Epithelial Cells
Source: PLoS One. 2011 Oct 18;6(10):e25562. doi: 10.1371/journal.pone.0025562 (PMC3196493; doi:10.1371/journal.pone.0025562)
Supplement: Data S1 — Additional information on methodology and results. (DOC) [file pone.0025562.s004.doc]

Submitted to Plos One, version: 20^th^ September 2011

**Supplementary Information**

**Materials and Methods S1**

**Preparation of exfoliated epithelial cells:** We have compared three techniques to optimize exfoliated epithelial cell isolation. First, our previously described technique (Kaeffer et al., 2007) with isolation through percolation on a column, the Percoll density gradient allowing to isolate cellular sheet at the top of the gradient and small clumps of cells or single-cell along the gradient or at the bottom of it, then the last technique described in this paper (**Figure 1A** and **B**). Different fixation techniques were also compared: Carnoy, paraformaldehyde 4%, formaldehyde 4% overnight with permeabilization by ethanol 70%. Carnoy fixative was unsuitable to detect membrane-labeling. The best preservation was obtained with formaldehyde 4% overnight with permeabilization by ethanol 70%.

**Studies on preterm infants:**

Excel file of raw data (anonymous patient identification, clinical and biological parameters) is included under « **data-S2.xls** ».

**Rat pup studies:**

Rat pups were sacrificed at 13H00 and at 01H00 to study exfoliated cells at two different time points in stomach lumen and in vitro exfoliation from fragments of gastric wall after a 60-min incubation at 37°C and 5% CO_2_***.*** When separated from dams, our 6-d pups were kept in a box on a water bath to avoid excessive dehydration and maintain body temperature for 5 hours. Then they were allowed to feed over one hour before sacrifice by inhalation of CO_2_ and cervical dislocation, and rapid dissection to recover the stomach. Stomachs were all full of milk. Contents were recovered by soft massaging of the stomach and stored at -70°C until the preparation of exfoliated cells. Stomachs were used either for in vitro exfoliation after rapid rinsing in 10 ml PBS0 or for immunohistochemical examination. Under our hands, the 12-day old rat pups can receive an oral bolus before re-uniting with their mother to manipulate the induction of exfoliation.

**Histology on rat gastric tissues:**

Gastric tissues of rat pups were either fixed in formaldehyde solution, embedded in epoxy and sectioned before hematoxilin-eosine staining or embedded in resin for cryosections and stored at -70°C before immunofluorescent staining and confocal microscopy examination. In order to realize immunostaining for microscopic examination**,** gastric sections were rapidly thawned and saturated for one hour at 4°C in PBS0 + 0,2% Bovine Serum Albumin fraction V (BSA). Primary antibodies were diluted in PBS0 + 0,2% BSA and incubated on sections overnight at 4°C in a special device. After 3 cycles of washing by PBS0, sections were incubated with secondary antibodies and Hoechst 33258.

**Immunocytochemistry and confocal imaging:**

Exfoliated cells were fixed in PBS0 with 4% formaldehyde overnight at 4°C to preserve the cell morphology. The next day, fixation medium was replaced with 70% ethanol for cell permeation. Samples were rehydrated by replacing ethanol with PBS0 with 0.2% bovine serum albumin (Fraction V, Sigma). Alternatively, exfoliated cells were fixed in Carnoy (ethanol : acetic acid / 3:1) and rehydrated before labeling or fixed in Paraformaldehyde 4%, and stored at 4°C. Cell phenotypes were analyzed by microscopic examination. Nuclear DNA was stained with Hoechst 33258 (Molecular Probes) or DRAC-5 (In Vitrogen) fluorochromes. Hoechst staining was systematically used to determine quiescent versus apoptotic nuclei. Apoptotic figures were rarely seen and not included in analysis*.* Cell and tissue preparations were mounted in Prolong Gold (In Vitrogen) and visualized under a Nikon Eclipse 400 microscope equipped for epifluorescence, Zeiss apotome microscope or Leica confocal microscope. The intensity of labeling by the primary – secundary antibodies complex was normalized by the total surface of the cellular body at the best plane of acquisition by densitometry with ImageJ software. Cell morphology was compared with buccal epithelial cells of human adult exfoliated by swabbing or a colon cancer cell line (Caco-2 cells).

**Statistics and Database:** There is no consensus to analyze rhythms, however we have used the methodology of Exploratory Data Analysis and TSA Serial Cosinor software (Expertsoft technologies, Esvres, France). Adjustment of periodic signal on these data by TSA Serial Cosinor indicates periodic signal by using exploratory data analysis principles (<http://www.itl.nist.gov/div898/handbook/index.htm>) as data are not equidistant in time due to slight variation (between 15 min to 45 min) in the timing of clinical samples.

**Results S1**

**Kinetics of exfoliation of preterm infants over two 24-hour cycles:** Serial collection of aspirates over 24-hours was performed to determine whether any fluctuation occurred in the rate of cellular loss in infants fed with a regular milk formula, and verify that exfoliation did occur even in infants fed intravenously and without any enteral feeding (**Figures 1 and S1**). As shown on Figure 1, oscillations are reproduced for 3 infants over 2 consecutive nights. The oscillation of CLOCK expression in the nuclei of buccal cells recovered by mechanical exfoliation can be compared with these data as to the hours of acrophase and bathyphase.

**Rat pup studies:**

The selection of optimal conditions to explore in vitro exfoliation from the stomach of rat pups was done on histological sections by recording the glandular architecture after 0 , 30, 60 and 90 min of tissue survival in culture medium at 37°C under 5% CO_2_. The gastric architecture was well-preserved after 60 min of incubation comparatively to 0 and 30 min. Moreover, the amount of exfoliated cellular material was visually higher than at 30 min both for 6-day and 12-day rat pups. The group of samples examined at 90 min revealed a beginning of tissue alteration. These results are similar to the description made on adult rats by Aoyama et al (2008).

The detection of H+/K+ ATPase-positive exfoliated cells in preparations of gastric contents was difficult to perform by microscopic examination and only rare epithelial cells with intact nuclei and membrane-bound labeling by anti-H+/K+-ATPase antibody were observed (results not shown). Thus we resorted to ELISA to evaluate the amount of antigens recovered by our isolation procedure in gastric contents of rat pups receiving or not a bolus before refeeding and sacrifice (**Figure S2**, white symbols). In the following experiments, we used 12-day old pups because they can easily receive per os a bolus of 500 µL saline solution before re-feeding by the mother allowing repeated nutritional manipulation. Gastric glands were maintained 60 min in culture medium containing 1 nmol/L MitoTracker Far Red to ascertain cellular viability of cells exfoliated in supernatants as well as cells remaining within the gastric glands. The amount of cellular material lost in supernatants was evaluated after centrifugation by two experimenters. The double blind visual inspection gave observations consistent with ELISA results presented on **figure S2** (black symbols). Mucus and cellular material lost by gastric glands at 13:00, as well as antigens detected in these samples by ELISA, were higher than the ones observed at 1:00.

By ELISA, the amount of antigens lost in the supernatants by cultured gastric glands (**Figure S2**, black symbols) as well as in gastric contents (**Figure S2**, white symbols) were significantly higher for every biomarker on rats sacrificed at 13:00 than for rats sacrificed at 01:00. A significant difference was found between 13:00 and 01:00 series for all biomarkers by Student's t-test (p<0.05) except for LC-3-b in contents, and CLOCK both in culture supernatants and contents. As all exfoliated cells are not of epithelial origin, we resorted to immunofluorescent microscopy to be able to identify single cell positively labeled by membrane-bound anti-H+/K+-ATPase antibody and harboring a quiescent nucleus at the surface of gastric gland or in cell culture supernatants.

On histologic sections or on exfoliated cells spotted onto slides, we selected exfoliated cells (**Figure S3 A)** and cells at the surface of the gland showing a quiescent nucleus (by Hoechst staining) and a membrane-bound labeling with anti-H+/K+ ATPase antibody. These gastric cells were also labeled for the expression of either Survivin, MAP LC-3-b or CLOCK (**Figure S3 B – D**). By quantification of MitoTracker Far Red, we found that both exfoliated cells (**Figure S3 E**) and cells at the surface of the gastric glands were viable (**Figure S3 F**).

To explore the putative circadian rhythm of exfoliation, we performed colocalization analysis to visualize an accumulation of CLOCK transcription factor within the cell nuclei of rat pups sacrificed at 13:00 or 01:00 (results not shown). Colocalization of CLOCK and of DNA in cells at the surface of the gastric glands showed a nuclear localization of CLOCK at 01H00, and an absence of nuclear localization at 13:00. Rat pups display a fully organized circadian physiology at D-30 (Sladek et al., 2007. Postnatal ontogenesis of the circadian clock within the rat liver. Am J Physiol Regul Integr Comp Physiol. 292:R1224-9) and before weaning (D-20) rat pups are believed to follow a diurnal rhythm. These observations are thus coherent with the diurnal oscillation of CLOCK found on preterm infants and a human adult volunteer.

**Figure S1. Kinetics of exfoliation of H+/K+-ATPase-positive cells over 24-hours followed by ELISA.** Four infants were sampled every 3 hours on two consecutive 24-h cycles. Note that infant 4 was unfed the first nycthemer of sampling (orange diamond ) but showing low level of cellular loss. Profiles were obtained in parallel preparations of H+/K+-ATPases detection, with antibodies against survivin (A, B), MAP-LC-3-b (C, D)), Pouf5-F1-Oct4 (E, F), PERIOD1 (G, H) and CLOCK (I, J). Note that by ELISA, we detect the antigens from any cellular phenotypes in the preparation along with cell-free antigens.

| 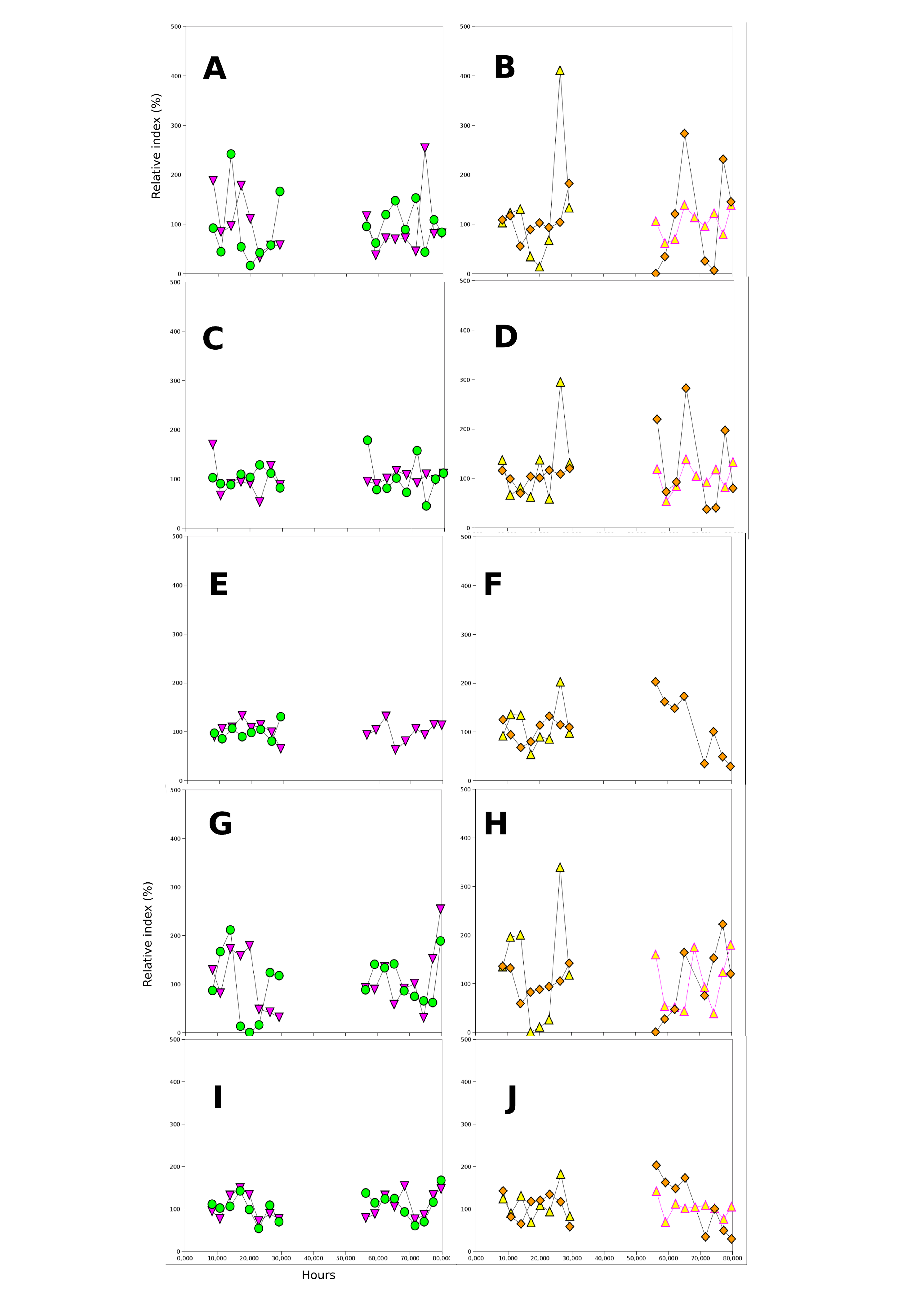 |
| --- |

**Figure S2. Detection of biomarkers of exfoliated cells in cell culture supernatants or stomach contents.** The detection of biomarkers was done by ELISA on preparations of epithelial gastric cells exfoliated from gastric glands maintained in culture for 60 min (black symbols) or recovered from gastric contents (white symbols) of 12-day-old rat pups refed one hour before sacrifice. Note the similarity of antigen detection profiles of black and white symbols for all biomarkers. A significant difference was found between 13:00 and 01:00 series for all biomarkers by Student't test (p<0.05) except LC-3-b in contents, and CLOCK both in vivo and in vitro exfoliation.

| 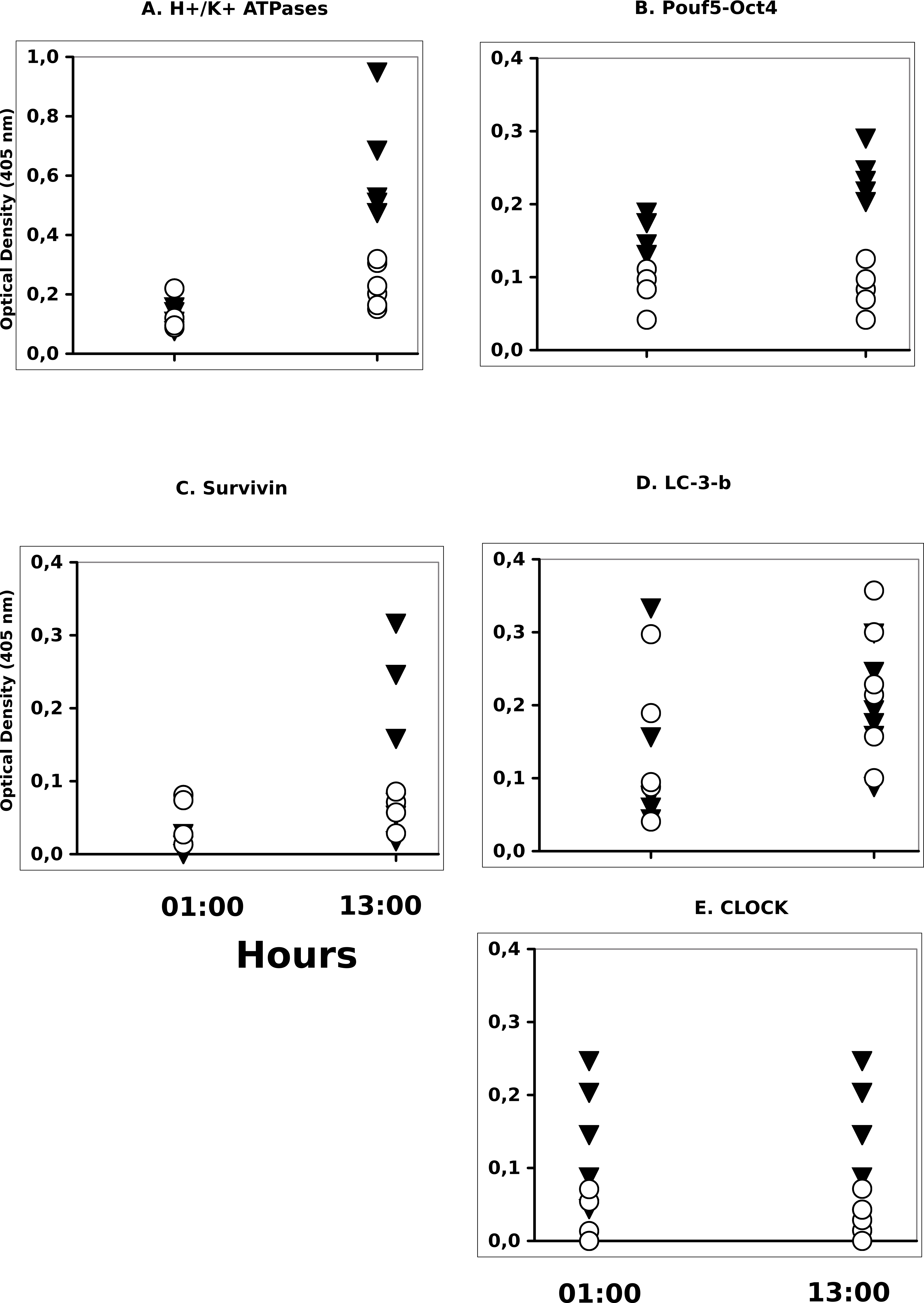 |
| --- |

Figure S3. Gastric exfoliated cells of rat pups. A. Typical exfoliated cells expressing membrane-bound H+/K+ ATPase (green, Alexa 488), showing a quiescent nucleus (blue, Hoechst). B. Survivin (red, Alexa 568). C. LC-3-B (red, Alexa 568). D. CLOCK (red, Alexa 568). E. Viable mitochondrial network in an exfoliated cell (yellow, MitoTracker Far Red). F. Surface epithelial cells of the gastric gland with viable mitochondries (yellow) showing a gradient of MitoTracker Far Red labeling with a maximum on the outside of the gastric sample and a minima inside the gastric sample.White bars stand for 10 µm.

| 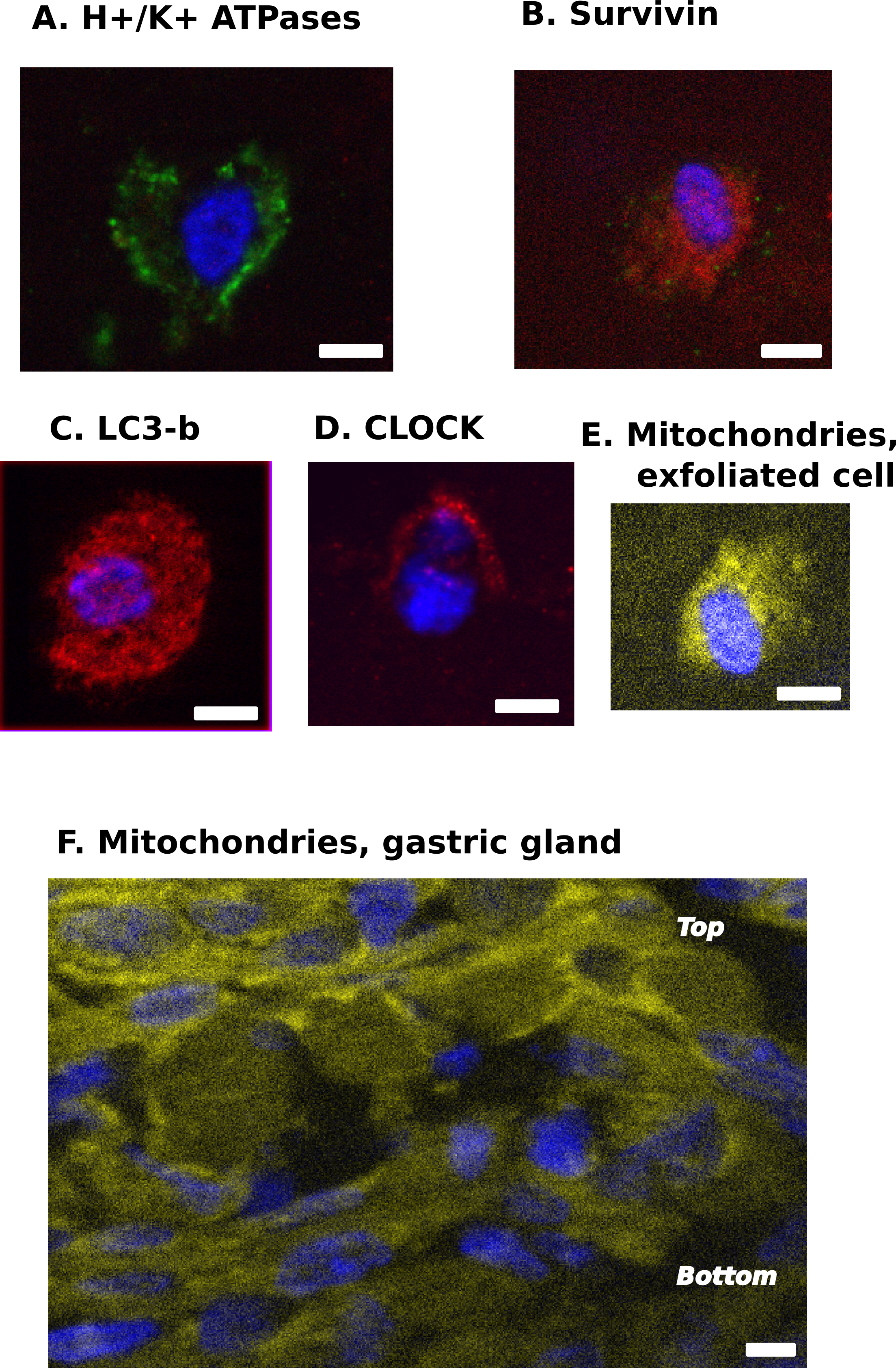 |
| --- |
